# Case Report: Acute Intracardiac Thrombosis in Children With Coronavirus Disease 2019 (COVID-19)

**DOI:** 10.3389/fped.2021.656720

**Published:** 2021-06-25

**Authors:** Hamid Bigdelian, Mohsen Sedighi, Mohammad Reza Sabri, Bahar Dehghan, Chehreh Mahdavi, Alireza Ahmadi, Mehdi Ghaderian, Hamid Rahimi, Atefeh Sadeghizadeh, Monirsadat Emadoleslami, Seyed Nasser Mostafavi, Rana Saleh, Niloofar Javadi, Maryam Derakhshan, Zahra Pourmoghaddas, Shima Sarfarazi Moghadam

**Affiliations:** ^1^Department of Cardiovascular Surgery, School of Medicine, Isfahan University of Medical Science, Isfahan, Iran; ^2^Pediatric Cardiovascular Research Center, Cardiovascular Research Institute, Isfahan University of Medical Sciences, Isfahan, Iran; ^3^Department of Anesthesiology and Pain Medicine, Pain Research Center, Iran University of Medical Sciences, Tehran, Iran; ^4^Department of Pediatrics, School of Medicine, Isfahan University of Medical Sciences, Isfahan, Iran; ^5^Student Research Committee, School of Medicine, Isfahan University of Medical Sciences, Isfahan, Iran; ^6^Department of Pathology, School of Medicine, Isfahan University of Medical Science, Isfahan, Iran

**Keywords:** COVID-19, acute thrombosis, children, cardiac surgery, case series

## Abstract

We herein describe a case series of children with SARS-CoV-2 infection (COVID-19) complicated with acute intracardiac thrombosis. The diagnosis of COVID-19 was confirmed through the reverse transcription-polymerase chain reaction (RT-PCR). Transthoracic echocardiography of patients revealed large intracardiac mobile masses resected successfully via cardiac surgery. The underlying mechanisms of this thrombus in the COVID-19 infection may be attributed to the hypercoagulation and inflammatory state of the disease incurred by the SARS-CoV-2 virus.

## Introduction

Coronavirus disease 2019(COVID-19) caused by a severe acute respiratory syndrome coronavirus 2 (SARS-CoV-2) was reported for the first time in Wuhan, Hubei, China in December 2019 and has spread rapidly throughout the world (1). According to the daily report of the World Health Organization (WHO), the epidemic of COVID-19 so far registered 2,751,166 cases and 76,936 deaths in Iran (2). However, epidemiological and clinical patterns of the COVID-19 remain largely unclear, particularly among pediatric patients. Children with COVID-19 have their specific clinical features and therapeutic responses but clinical manifestations of this disease in children might be less severe (3). Although COVID-19 is recognized as an acute respiratory tract infection, accumulating clinical data indicates cardiovascular complications of the disease, including myocarditis, arrhythmias, cardiogenic shock, and acute myocardial injury (4). More importantly, cardiovascular thrombosis due to COVID-19-induced hypercoagulopathy is a serious and life-threatening complication that required emergency intervention in some cases (5, 6). To our knowledge, hypercoagulopathy associated with COVID-19 in children has been rarely reported. Here, we report three COVID-19 children with acute intracardiac thrombosis treated with cardiac surgery.

## Case 1

**Figure 1:**
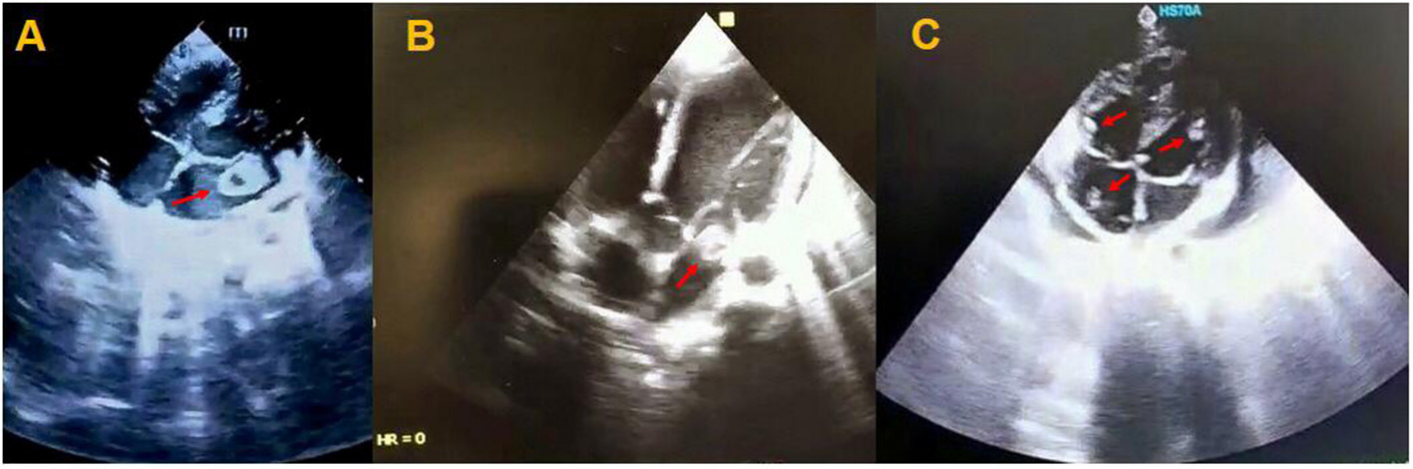
Transthoracic echocardiogram of patients showing a mobile mass in the left atrium with attachment to the posterior leaflet of the mitral valve **(A)**, a large mobile mass in the left atrium **(B)**, and multiple mobile homogenous masses in the right side of the heart and left ventricle **(C)**.

An 11-year-old girl was admitted to the Emergency Department of Pediatric Medical Center with fever (39°C), coughing, tachypnea (38 times per minute), low oxygen saturation (93% in room air), and decreased level of consciousness (LOC). Glasgow Coma Scale (GCS) was 13 and lower extremities rashes were found in her physical examination. Also, results of the blood test on admission time revealed leukocytosis and increased level of C-reactive protein (CRP) (Table 1). The patient was transferred to the pediatric intensive care unit (PICU) and nasopharyngeal swab and stool sample analysis by reverse transcription-polymerase chain reaction (RT-PCR) confirmed COVID-19 infection and, therefore, drug treatment for the COVID-19 was started according to the Iranian pediatric protocol, including hydroxychloroquine (5 mg/kg/dose), lopinavir /ritonavir (230 mg/m^2^/dose), ceftriaxone (75 mg/kg/dose), and vancomycin (10 mg/kg/dose) (7). Because of persistent fever and tachycardia, pediatric cardiology consultation was requested and done on the 3rd day of hospitalization showing left ventricular ejection fraction (LVEF) of 64%, mild mitral regurgitation (MR), trivial tricuspid regurgitation (TR), trivial pulmonary insufficiency (PI), and a large mobile mass in the left atrium (LA) with attachment to the posterior leaflet of the mitral valve (Figure 1A). Hence, she became a candidate for emergency surgical intervention and cardiac surgery was performed on the 5th day. Intraoperative observations revealed a large thrombus in the LA with necrosis of the posterior leaflet of the mitral valve. Left atrium thrombus was resected completely and the mitral valve was repaired. Her clinical conditions improved following surgery and the results of postoperative echocardiography showed a normal heart function. She was discharged in good health condition after 10 days of hospitalization with negative RT-PCR for COVID-19.

**Table 1:** Laboratory test results of patients at the time of admission.

**Variables**	**Reference range**	**Case 1**	**Case 2**	**Case 3**
RBC count (×10*12/L)	4.5–6.5	3.75	3.89	3.79
Hematocrit (%)	41–51	32.9	34.2	33.9
Hemoglobin (g/dl)	13–17	11.8	11	11.5
WBC (×10*9/L)	4.5–11	16.3	14.2	12.3
Lymphocyte count (%)	20–40	10	15	27
Platelet count (×10*9/L)	150–450	316	219	252
ESR (mm/h)	0–20	56	80	22
CRP (mg/L)	Up to 6	29	38	11
PT (s)	11–13	13	14.1	14
PTT (s)	26–45	38	38	34
INR	0.9–1.2	1	1.2	1.2
D-dimer	Up to 500	490	530	2,000
Blood culture (3 × )	±	–	–	–
RT-PCR SARS-CoV-2	±	+	+	+

*RBC, red blood cell; WBC, white blood cell; ESR, erythrocyte sedimentation rate; CRP, C-reactive protein; PT, prothrombin time; PTT, partial thromboplastin time; INR, international normalized ratio*.

## Case 2

**Figure 2:**
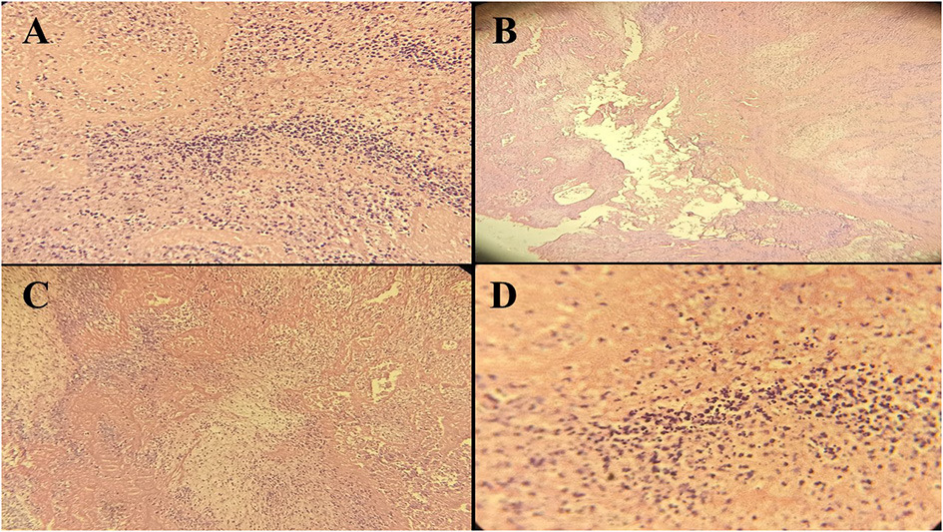
Histopathological manifestations (40x resolution) of the posterior leaflet of mitral valve tissue in case 2 showing fibrinoid necrosis and cellular debris **(A)**, fibrinoid necrosis without evidence of bacterial colonization **(B)**, dense neutrophilic infiltration with cellular debris **(C)**, and necrotic area with neutrophilic debris induced by SARS-CoV-2 **(D)**.

An 7-year-old girl was referred to the Emergency Department of Pediatric Medical Center with fever (38.2°C), tachypnea (36 times per minute), lower extremities rashes, abdominal pain, and bilious vomiting. She had no significant past medical history, but she had close contact with a COVID-19 patient. In physical examination, tachycardia, and low blood pressure were observed, and thus, she was transferred to the PICU for respiratory support by continuous positive airway pressure (CPAP) along with pharmacotherapy. Leukocytosis with increased CRP and erythrocyte sedimentation rate (ESR) were found in blood test (Table 1) and nasopharyngeal swab analysis by RT-PCR confirmed COVID-19. According to the cardiorespiratory and gastrointestinal symptoms and laboratory results, early diagnosis of COVID-19-induced multisystem inflammatory syndrome in children (MIS-C) was made and pharmaceutical treatment, including hydroxychloroquine (5 mg/kg/dose), lopinavir/ritonavir (230 mg/m^2^/dose), intravenous immunoglobulin (IVIG /2 gr/kg), methylprednisolone (30 mg/kg), and enoxaparin (2 mg/kg per 12 h) was started. Pediatric cardiology consultation was requested because of cardiomegaly and pulmonary edema in her chest X-ray and transthoracic echocardiography showed LVEF of 69% with better LV systolic function, minimal pericardial effusion, mild TR, trivial PI, and a large mobile mass in the LA (Figure 1B). Consequently, she underwent cardiac surgery on the 6th day of hospitalization and intraoperative observations revealed multiple large thrombi in LA and right atrium (RA) with necrosis of posterior leaflet of the mitral valve. Intracardiac thrombi were removed completely and the mitral valve was repaired. Second echocardiography after surgery showed normal heart function and she was discharged from hospital after 2 weeks in good health condition with negative COVID-19 test. In the histopathological assessment, fibrin clot, fibrinoid necrosis, and surface thrombus with dense neutrophilic infiltration were found in the necrotic tissue of posterior leaflet of the mitral valve (Figures 2A–D).

## Case 3

An 8-year-old girl was referred to the Emergency Department of Pediatric Medical Center with fever (38.2°C), tachycardia (PR 135), and tachypnea (40 times per min). Past medical history of the patient revealed that she had undergone orthopedic surgery a couple of weeks ago because of car accident injures. In her blood test, leukocytosis, increased CRP, and a remarkable rise in D-dimer were found and RT.PCR test for COVID-19 was positive (Table 1). Primary diagnosis of pulmonary thromboembolism (PTE) was made and pharmaceutical treatment, including hydroxychloroquine (5 mg/kg/dose), methylprednisolone (30 mg/kg), enoxaparin (2 mg/kg per 12 h), lopinavir /ritonavir (230 mg/m^2^/dose) was started. On the 2nd day of hospitalization, echocardiography was performed showing LVEF of 68%, mild LA and LV enlargement, trivial TR, trivial PI, and multiple homogenous masses in the LV and right side of the heart as well as clot strands in the main pulmonary artery (MPA) (Figure 1C). Therefore, she underwent emergency cardiac surgery on the 3rd day and multiple thrombi were resected from the cardiac chambers with the repair of the tricuspid valve. Also, blood clots were removed successfully from the MPA through thrombectomy. She was admitted to the PICU and pediatric ward and was discharged from the hospital after 2 weeks. Second echocardiography indicated normal cardiovascular function and she was discharged from the hospital in good health condition after 15 days with a negative test for COVID-19.

Pathological assessment of specimens in three children showed no fungal and bacterial infection and also SARS-CoV-2 RNA was not detected in the thrombi.

## Discussion

Among the COVID-19 patients, the notifiable cases are adults complicated with fever, dry cough, fatigue, and worsening dyspnea, whereas pediatric cases are less reported in the literature and clinical profiles of those children have not been well-defined (8). A prior study in our country reported that fever is more prevalent in COVID-19 children aged ≥5 years compared to the patients aged <5 years and nearly one-third of children experience diarrhea and/or vomiting (3). We herein report three COVID-19 children complicated by acute intracardiac thrombosis treated with cardiac surgery. Although COVID-19 has been known as an acute respiratory disease affecting respiratory tract by causing pneumonia and acute respiratory distress syndrome (ARDS), recent reports have indicated that COVID-19 is associated with a prothrombotic state that can lead to the microvascular, venous, and/or arterial thrombosis (9). A prior study by Panigada et al. reported an increased risk of venous thrombosis in COVID-19 patients due to hypercoagulability induced by inflammation (6). Another study involving 184 COVID-19 patients reported CT angiography and/or ultrasonography confirmed venous thromboembolism in 27% of patients and arterial thrombotic events in 3.7% of patients. Also, pulmonary embolism was the most frequent thrombotic event and may be massive with significant RV failure (10).

It has been well-documented that the innate immune response and thrombotic response are closely linked and increased levels of acute-phase reactants such as fibrinogen and CRP may contribute to the hypercoagulable state associated with COVID-19, increasing the severity of disease (11, 12). Furthermore, COVID-19-induced inflammation plays a pivotal role in cardiovascular complications where IL-6, together with other cytokines, establishes a prothrombotic state via disabling the natural inhibitors of hemostasis. Dysfunction of endothelial cells induced by COVID-19 infection leads to excess thrombin generation and fibrinolysis shutdown. The hypoxia observed in severe COVID-19 can also stimulate thrombosis via increasing blood viscosity. Moreover, activation of the hypoxia-inducible factor-1 (HIF-1) signaling pathway increases the coagulability of the blood (13). The intracardiac thrombus formation in COVID-19 children has rarely been reported and our findings support current reports indicating higher thrombotic risk in COVID-19 patients (9). The presence of a large intracardiac thrombus was an unexpected finding in our cases because they had no history of heart disease or coagulopathy. Resection of such a large thrombus in our patients needs emergency surgery in the absence of any contraindication for surgery. Besides, anticoagulation treatment correlates with the theoretical risk of lysed clots lodging in pulmonary arteries and consequent PTE (14).

To sum up, there is an increasing concern about hypercoagulopathy state in COVID-19 patients, especially children. Therefore, conservative treatment with anticoagulation along with vigilant workup is advised in all COVID-19 patients to prevent subsequent hypercoagulable states.

## Data Availability Statement

The original contributions generated for the study are included in the article/supplementary material, further inquiries can be directed to the corresponding author/s.

## Ethics Statement

This study was reviewed and approved by the Medical Research Ethics Committee at Isfahan University of Medical Science, Isfahan, Iran. Written informed consent was obtained from the patient's next-of-kin for the publication of this research.

## Author Contributions

All authors contributed to the analysis, interpretation of data, wrote the manuscript, approved the final version of the manuscript, and agreed to be accountable for all aspects of the work.

## Conflict of Interest

The authors declare that the research was conducted in the absence of any commercial or financial relationships that could be construed as a potential conflict of interest.
